# Clinical Audit in the Elderly by Assessing Hydration and Renal Functions Before and After Contrast CT Scans at District Headquarters Hospital, Dera Ismail Khan, Pakistan

**DOI:** 10.7759/cureus.67661

**Published:** 2024-08-24

**Authors:** Rohail Razzaq, Muhammad Hamza Khan, Seema Shaheen, Najeeb Ullah

**Affiliations:** 1 Burn Unit, Mufti Mehmood Memorial Teaching Hospital, Dera Ismail Khan, PAK; 2 Pediatrics Department, Mufti Mehmood Memorial Teaching Hospital, Dera Ismail Khan, PAK; 3 General Medicine Department, Mufti Mehmood Memorial Teaching Hospital, Dera Ismail Khan, PAK; 4 Internal Medicine Department, Rehman Medical Institute, Peshawar, PAK

**Keywords:** contrast-induced nephropathy, clinical audit, contrast-enhanced computed tomography, district head quarters, urea and electrolytes, computerized tomography scan, elderly people, hydration status, renal function tests, quality improvement projects

## Abstract

Introduction

Contrast-induced nephropathy (CIN) is a serious risk involved in computed tomography (CT) scans, particularly for older people. The main idea of this clinical audit was to assess current practices regarding renal function tests (RFTs) and hydration status before and after contrast CT scans in older patients at District Headquarters Hospital (DHQ), Dera Ismail Khan, Pakistan, and to implement recommendations for improvement. CIN is a form of acute kidney injury that occurs after the administration of contrast dye used in imaging procedures and is characterized by a sudden deterioration in renal functions.

Methods

This clinical audit checked adherence to renal protection protocols in elderly patients undergoing contrast CT scans. Conducted over three cycles from July 5 to August 15, 2022, this clinical audit included 30 patients aged 75 and above. Each cycle had 10 patients, divided equally between males and females, and further categorized into age groups of 75-85 years and 86-95 years. Data collection involved reviewing patient files, medication charts, and CT scan reports. Compliance with RFT documentation and hydration before and after the CT scan was assessed against the standards set by Basildon and Thurrock University Hospitals NHS Foundation Trust. Data were analyzed using Microsoft Excel 2023 (Microsoft® Corp., Redmond, WA), and graphs were created using Microsoft Word 2023 (Microsoft® Corp., Redmond, WA).

Results

The mean age ± standard deviation (SD) for males was 81.8 ± 5.01 in the first cycle, 83.4 ± 6.46 in the second cycle, and 82.4 ± 4.72 in the third cycle. For females, the mean age ± SD was 83.2 ± 5.80 in the first cycle, 85.2 ± 6.41 in the second cycle, and 83.0 ± 6.12 in the third cycle. The first audit cycle revealed that, while all patients (100%) had their RFTs documented before the CT scan, only 20% were adequately hydrated pre-scan, and none (0%) had RFTs performed post scan. Post-scan hydration was also low at 20%. These findings highlighted gaps in adherence to renal protection protocols. The second cycle showed improvements, with pre-scan hydration adherence increasing to 80%, post-scan RFTs to 60%, and post-scan hydration to 70%. By the third cycle, full compliance (100%) was achieved across all standards, including pre- and post-scan renal functions test and hydration.

Conclusion

The clinical audit at District Headquarters Hospital, Dera Ismail Khan, addressed gaps in renal protection protocols for elderly patients undergoing contrast CT scans. The audit improved adherence over three cycles through targeted interventions, including staff training, implementation of checklists, patient education, modifying the reporting format, and providing instructions in the local language. It also highlighted the importance of continuous education and regular monitoring. The clinical audit would be expanded to another hospital within the medical teaching institute, Dera Ismail Khan. This measure will maintain and enhance patient care, prevent CIN, and improve the renal health of elderly patients.

## Introduction

A computed tomography (CT) scan is used in radiology for imaging studies and was pioneered by physicist Allan MacLeod Cormack, an electrical engineer Godfrey Hounsfield, and a neuro-physician James Ambrose, who demonstrated its vast clinical significance [1,2]. CT scans with contrast involve the injection of iodine-based contrast dye into the bloodstream, which enhances the visibility of structures. At the same time, non-contrast CT does not include injection of contrast dye in the bloodstream [3]. After intravascular contrast administration, contrast-induced nephropathy (CIN) reduces kidney filtration function. It is the third leading cause of acute renal failure in healthcare centers, accounting for 12% of cases [4]. The mechanism of action of CIN is established on three processes: medullary ischemia, formation of reactive oxygen species, and direct tubular cell toxicity [5]. The Kidney Disease Improving Global Outcomes (KDIGO) defines CIN as a rise in serum creatinine by 26.5 mmol/L within 48 hours or a rise in serum creatinine by 1.5 times from the initial level within one week or urine output less than 0.5 mL/kg/hour of body weight for more than six continuous hours after exposure to a contrast agent [6]. The serum creatinine reaches its highest levels three to five days after contrast media administration and typically returns to normal within one to three weeks [7]. CIN is associated with increased hospital stay in people who are at risk [7,8]. Risk factors for CIN include diabetes, chronic renal failure, hypotension, and age [9]. The hazard of CIN in older people (≥ 75 years) is 7.5% [9]. To alleviate the associated risk, it is essential to ensure that patients are hydrated, and their renal function is assessed before and after contrast administration. The age was selected as a risk factor for this audit (age ≥ 75 years), and these elderly were checked to determine whether their renal functions were ordered and whether they were hydrated before and after the contrast CT scan. The main aims of this clinical audit were to check the current practices regarding renal function tests (RFTs) and hydration status before and after the contrast CT scans in elderly patients and to implement recommendations to improve these practices.

## Materials and methods

Study design, setting, and duration

This clinical audit was conducted at District Headquarters Hospital Medical Teaching Institute, Dera Ismail Khan, Pakistan, over three cycles from July 5 to August 15, 2022.

Sample size

Thirty elderly patients were selected for this clinical audit, with each loop consisting of 10 patients evenly distributed into elderly males and females. This clinical audit focused on elderly patients aged 75 and above undergoing elective contrast CT scans, either admitted or outpatients, at District Headquarters Hospital, Dera Ismail Khan. Emergency CT scans and patients younger than 75 were excluded from this audit. Furthermore, the elderly male and female patients were divided into age groups (75-85 years) and (86-95 years). In the first cycle (n=10), there were three males and two females aged between 75 and 85 years and two males and three females aged between 86 and 95 years. In the second cycle (n=10), there were two males and three females aged between 75 and 85 years and three males and two females aged between 86 and 95 years. The third cycle (n=10) included three males and three females aged between 75 and 85 years and two males and two females aged between 86 and 95 years (Table 1).

**Table 1:** Age distribution and gender ratio across three audit cycles for elderly patients who underwent elective contrast CT scans This balanced age and gender distribution minimizes demographic biases and ensures the reliability and validity of the audit results. SD: standard deviation

S. No	Age Group	1st Cycle (n=10)		2nd Cycle (n=10)		3rd Cycle (n=10)	
		MALE (n=5)	FEMALE (n=5)	MALE (n=5)	FEMALE (n=5)	MALE (n=5)	FEMALE (n=5)
1.	75-85 years	3	2	2	3	3	3
2.	86-95 years	2	3	3	2	2	2

Sample collection

For the first cycle, data were collected from the general medicine department's request paper, the medication chart of patient files, and reports of CT scans from the radiology department. Ten patients were selected for this cycle, and information on whether RFTs were performed and hydration status was recorded. The data were collected in the same way as the initial audit cycle for the second and third cycles.

Data analysis

Data were analyzed using Microsoft Excel 2023 (Microsoft® Corp., Redmond, WA), and graphs were made using Microsoft Office Word 2023 and Microsoft Excel 2023. Grammar was checked on the Grammarly software application.

Standard

We conducted the audit according to the guidelines published by Basildon and Thurrock University Hospitals NHS Foundation Trust in September 2012 [10]. These guidelines identify patients at risk of CIN, outline pre-contrast care, manage contrast media risks, and provide post-contrast care instructions. The standards selected included documenting recent RFTs before the contrast CT scan, ensuring pre-scan hydration, ordering post-scan RFT, and rehydrating patients after the CT scan, all with 100% compliance (Table 2).

**Table 2:** Standard selected from the guidelines published by Basildon and Thurrock University Hospitals NHS Foundation Trust RFT: renal function test; U&E: urea and electrolytes; CT: computed tomography

S. No	STANDARD	STANDARD COMPLIANCE RATE
1.	Recent RFT (U&E) is documented before the CT scan	100%
2.	The patient is hydrated with 1 liter of oral fluid/1 liter of 1.26% Sodium Bicarbonate/1 liter of 0.9% sodium chloride solution before CT scan	100%
3.	RFT (U&E) is ordered after contrast CT scan	100%
4.	The patient is hydrated again after the contrast CT scan	100%

Interventions

Training sessions were conducted for the training medical officers, medical officers, house officers, nurses, and paramedics of general medicine and radiology departments on the importance of conducting RFTs and maintaining proper hydration before and after the contrast CT scan. Additionally, current radiology CT reports were modified, including pre- and post-renal functions and hydration checklists (Table 3). 

**Table 3:** Core pre-contrast and post-contrast requirements in our up-to-date radiology reports GFR: glomerular filtration rate; RFT: renal function test; U&E: urea and electrolytes

S. No	Pre-contrast Requisites	Post-contrast Requisites
1.	"Are recent renal function tests (Urea, Creatinine, GFR, Electrolytes) documented for outpatients?"	Outpatients should return to the hospital within 48 hours of contrast CT for Renal function tests (Urea, creatinine, GFR, and Electrolytes)
2.	"Are recent renal function tests (Urea, Creatinine, GFR, Electrolytes) documented for admitted patients?"	For admitted patients, renal function tests (Urea, creatinine, GFR, Electrolytes) should be performed within 48 hours after contrast CT
3.	"Was hydration done with 1 liter of oral fluid for outpatients? (YES or NO)"	Outpatients advised to drink water at home after contrast, CT scan
4.	Was hydration administered with 1 liter of sodium bicarbonate or 1 liter of sodium chloride for admitted patients starting one hour before the contrast CT? (YES or NO)	For admitted patients, hydration should be continued with 1 liter of sodium bicarbonate or 1 liter of sodium chloride for 4 hours after the contrast CT scan

Better communication between radiology and the general medicine department was fostered to ensure close coordination in patient care. The radiology department staff was instructed to mention in reports of elderly patients that they should be hydrated and have their renal functions repeated after 48 hours of contrast.

The nephrology department was consulted for patients with deranged renal functions following contrast CT, which improved patient care.

Patients were educated on the importance of hydration before CT scans with contrast. Especially, older people who had no comorbidities were asked to take oral drinks before and after the scan.

A regular audit cycle will be conducted to check compliance with these guidelines. Furthermore, the medical teaching institute Dera Ismail Khan has two hospitals. We initially conducted this audit in the district headquarters hospital, Dera Ismail Khan. This audit will be expanded to Mufti Mehmood Memorial Teaching Hospital, Dera Ismail Khan.

Ethics

The institute does not require ethical approval for this study due to its retrospective nature. However, the study has been authorized by the Medical Director of the District Headquarters Hospital, Dera Ismail Khan.

## Results

The mean age ± standard deviation (SD) for males was 81.8 ± 5.01 in the first cycle, 83.4 ± 6.46 in the second cycle, and 82.4 ± 4.72 in the third cycle. For females, the mean age ± SD was 83.2 ± 5.80 in the first cycle, 85.2 ± 6.41 in the second cycle, and 83.0 ± 6.12 in the third cycle (Table 4).

**Table 4:** Mean age ± standard deviation (SD) for males and females across each cycle of the clinical audit SD: standard deviation

S. No	Mean Age ± SD	1^st^ Cycle (n=10)	2^nd^ Cycle (n=10)	3^rd^ Cycle (n=10)
1.	Male (n=5)	81.8 ± 5.01	83.4 ± 6.46	82.4 ± 4.72
2.	Female (n=5)	83.2 ± 5.80	85.2 ± 6.41	83.0 ± 6.12

Adherence with RFTs before contrast CT scans was five out of five for males and five out of five for females across all three cycles. This showed that all patients, regardless of gender, had their RFTs documented before the contrast CT scan. In the first cycle, only one out of five males and one out of five females were hydrated before the scan. In the second cycle, this adherence improved to four out of five for both genders. By the third cycle, five out of five male and female patients were hydrated before the contrast CT scan. Adherence with RFTs after contrast CT scans were zero out of five for both males and females in the first cycle. In the second cycle, adherence improved to three out of five for both genders. By the third cycle, five out of five adherence was achieved for the male and female patients. In the first cycle, only one out of five male and female patients received proper hydration after the scan. This adherence increased to three out of five for males and four out of five for females in the second cycle. By the third cycle, five out of five of both genders received hydration post-scan (Table 5).

**Table 5:** Adherence with the standards for contrast CT scans, focusing on renal function test (U&E) and hydration status before and after the scans in male and female elderly patients CT: computed tomography; U&E: urea and electrolyte; RFT: renal function test

S. No	Standard	1st Cycle (n=10)		2^nd ^Cycle (n=10)		3^rd^ Cycle (n=10)	
		Male (n=5)	Female (n=5)	Male (n=5)	Female (n=5)	Male (n=5)	Female (n=5)
1.	Renal Function Tests (U&E) before contrast CT scan	100% (5)	100% (5)	100% (5)	100% (5)	100% (5)	100% (5)
2.	Hydration Status before contrast CT scan	20% (1)	20% (1)	80% (4)	80% (4)	100% (5)	100% (5)
3.	Renal Function Tests (U&E) after contrast CT scan	0% (0)	0% (0)	60% (3)	60% (3)	100% (5)	100% (5)
4.	Hydration Status after contrast CT scan	20% (1)	20% (1)	60% (3)	80% (4)	100% (5)	100% (5)

**Figure 1:**
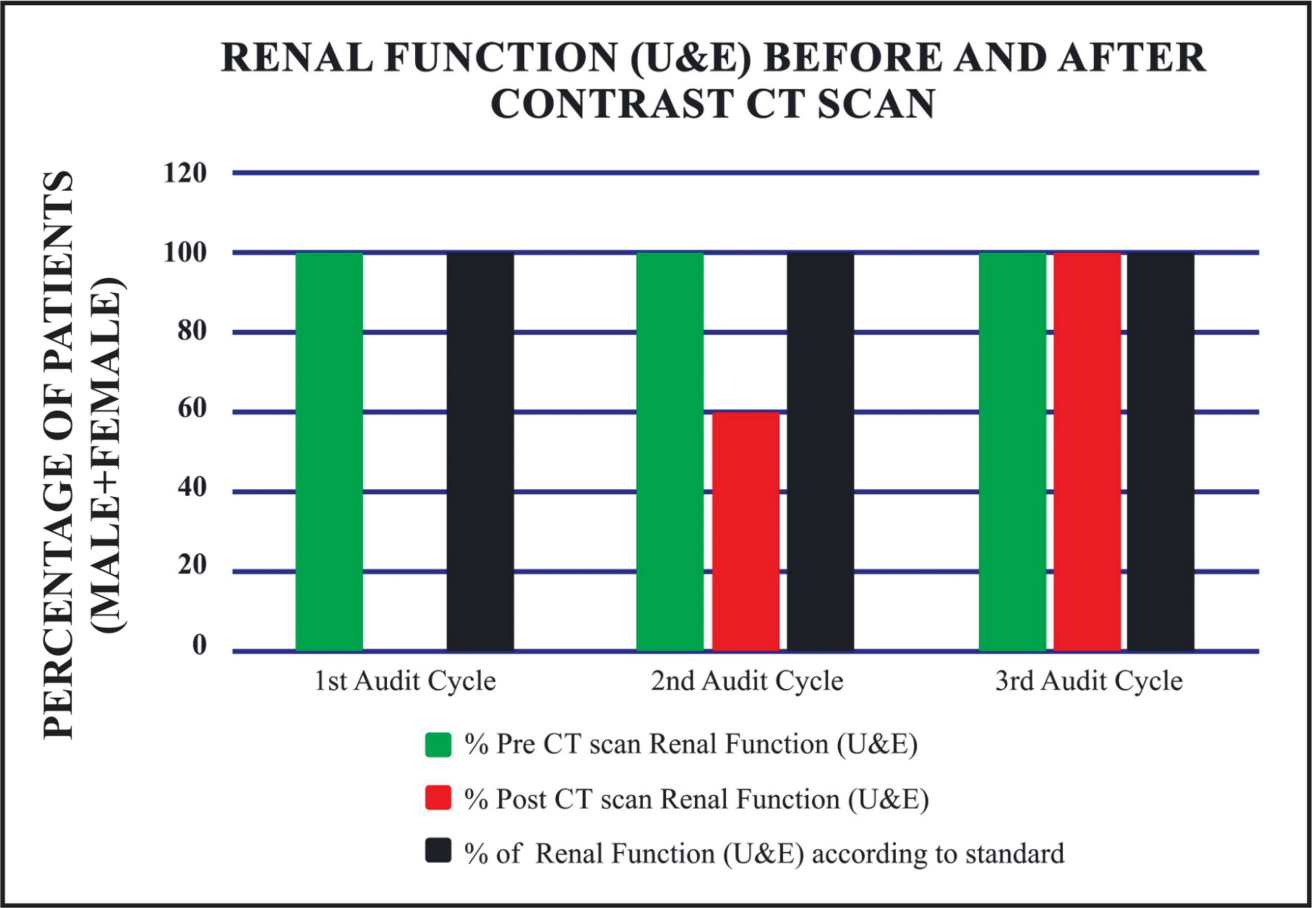
Renal function before and after contrast CT scans U&E: urea and electrolyte; CT: computed tomography

**Figure 2:**
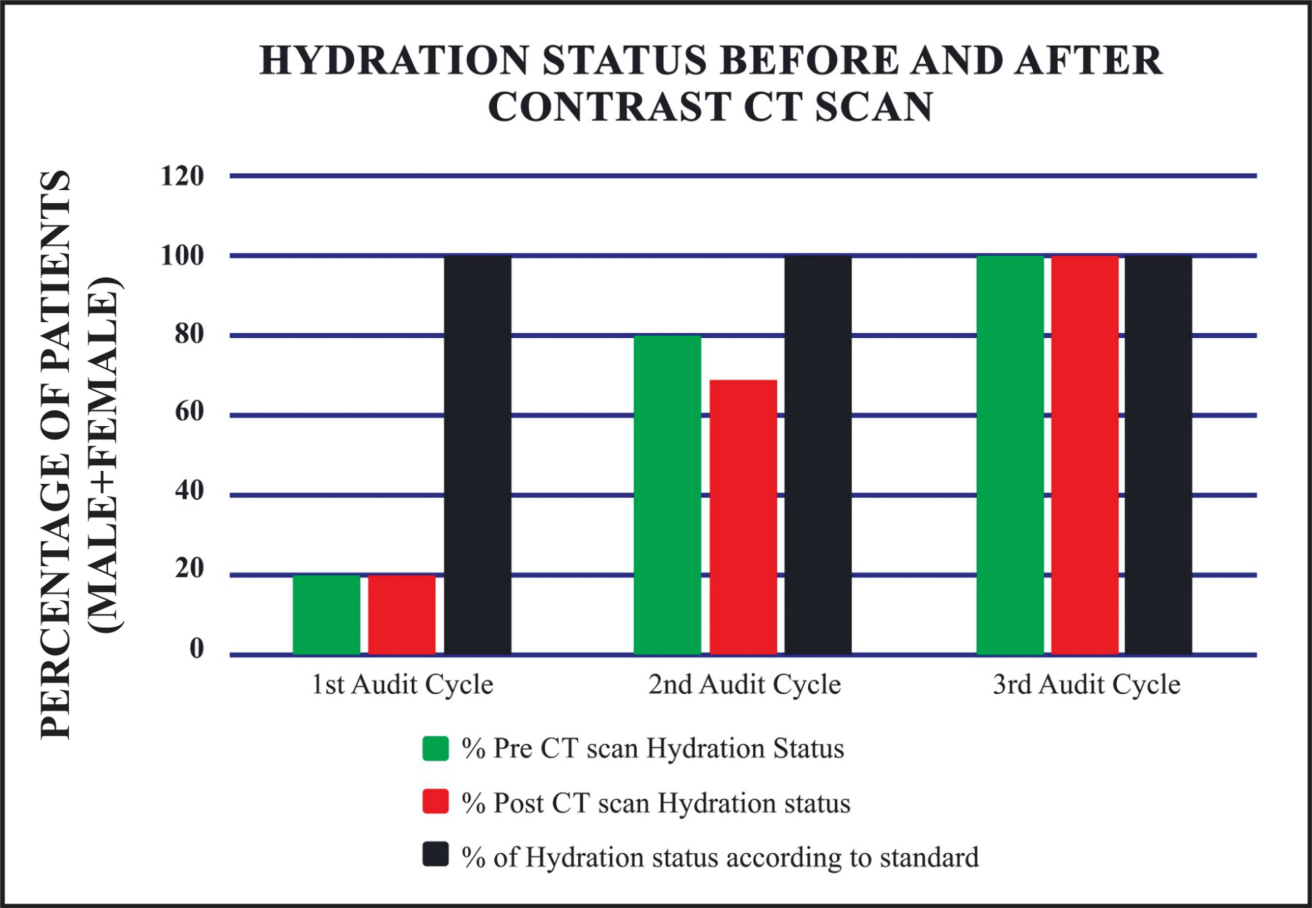
Hydration status before and after contrast CT scans CT: computed tomography

In analyzing the combined trends for males and females, the first cycle demonstrated full adherence (100%) with documenting pre-scan RFTs (Figure 1). However, adherence to hydration status before and after the scan was poor (20%), and post-scan RFTs were entirely neglected. The second cycle showed progress, with pre-scan hydration adherence rising to 80%, post-scan RFTs increasing to 60%, and post-scan hydration reaching 70% (Figure 2). By the third cycle, full adherence (100%) across all standards was achieved, including pre-scan RFTs, pre-scan hydration, post-scan RFTs, and post-scan hydration (Table 6).

**Table 6:** Combined trend analysis: adherence rates of standards followed for elderly patients (males and females % combined) at District Headquarters Hospital compared to Basildon and Thurrock University Hospitals NHS Foundation Trust RFT: renal function test; U&E: urea and electrolyte; CT: computed tomography; DHQ: district headquarters hospital; NHS: national health service

	Standard	Percentage of standard in DHQ Hospital, Dera Ismail Khan			Percentage of standard according to Basildon and Thurrock University Hospitals NHS Foundation Trust
S. No		1^st^ Cycle (n=10)	2^nd ^Cycle (n=10)	3^rd^ Cycle (n=10)	Standard (%)
1	Renal function tests (U&E) before contrast CT scan	100% (10)	100% (10)	100% (10)	100%
2	Hydration status before contrast CT scan	20% (2)	80% (8)	100% (10)	100%
3	Renal function tests (U&E) after contrast CT scan	0% (0)	60% (6)	100% (10)	100%
4	Hydration status after contrast CT scan	20% (2)	70% (7)	100% (10)	100%

## Discussion

The incidence of CIN in older adults (≥ 75 years) is 7.5%. CIN is the third leading cause of acute renal failure in healthcare facilities and is usually transient, and renal function returns to normal levels over time. One-third of patients develop some kidney issues, and 1% of people require dialysis. The incidence of dialysis remains high in patients with underlying conditions, such as chronic kidney disease and diabetes [11]. The most important approach is to take safeguarding measures because no definite treatment for CIN exists. Pre-procedural hydration is the most crucial stratagem to decrease the incidence of CIN [12,13]. Volume expansion opposes the adipose tissue renin-angiotensin-aldosterone system (RAAS), reduces the generation of vasoconstrictors and reactive oxygen species formation, and decreases the tubule-glomerular feedback mechanism [12-15]. A meta-analysis demonstrated that oral fluid intake with a specified quantity is as adequate as the venous (IV) route for CIN [15]. Hydration with isotonic saline solution (0.9% NaCl) is far better than half-isotonic saline (0.45% NaCl), most likely due to the prior's more remarkable ability to expand intracellular fluid volume [16]. There is no proof favoring the importance of sodium bicarbonate over sodium chloride. On the date of contrast computed tomography, the patient should receive one liter of sodium bicarbonate 1.26% intravenously over five hours, according to the guidelines published by Basildon and Thurrock University Hospitals NHS Foundation Trust. Ideally, the infusion should start one hour before the CT scan and continue for four hours after. If sodium bicarbonate 1.26% is unavailable, sodium chloride 0.9% can be used as an alternative. The trust guidelines also suggest that the most recent real function test (U&E) is documented, and post-CT scan RFTs (U&E) should be checked within 48 hours. There is no CIN risk if the renal function is at the initial value or has improved. If the renal function is abnormal, the RFT (U&E) should be repeated after 24 hours. If there is a 25% increase in serum creatinine, a referral should be made to a nephrology ward. According to Kanthasamy et al.'s clinical audit, all 30 patients had their renal function checked before the contrast; only 57% (n=17) had it rechecked within seven days of post-contrast, and only 53% (n=15) were hydrated [17]. A decrease in renal function was observed in 59% of the patients, with 23% developing CIN as per KDIGO criteria. Additionally, 75% of those who developed CIN were not hydrated pre- or post-procedure [17]. In our clinical audit, 100% (n=10) of patients had their RFTs checked before contrast in the first cycle, but 0% (n=0) had their RFTs performed post-contrast, while 20% (two out of 10) were hydrated in the first audit cycle after the contrast. This improved in the next audit loops. Their recommendations included a checklist, which we also introduced in our interventions. The longitudinal cohort study conducted by Aoki et al. [18] found that 64.5% of patients received some form of prophylactic measures before contrast-enhanced CT, with 49.5% receiving intravenous hydration with normal saline. Post-contrast, 47.3% of patients received some preventive care, primarily hydration, but 52.7% did not receive any preventive measures. Additionally, 45.16% of patients did not have their serum creatinine measured after the contrast. Only 54.8% had creatinine measurements taken before and after the contrast [18]. In our clinical audit, 100% of patients had their renal functions done before the contrast, 20% received pre-contrast hydration, 0 % had post-contrast RFTs, and 20% had post-contrast hydration done. By the third cycle, compliance reached 100% for pre- and post-contrast hydration and RFTs. Furthermore, the elderly in their clinical audit were divided into male and female elderly patients, just like our study, but the age they selected was 70 years, while ours was greater than equal to 75 years. A clinical audit on the prevention of acute kidney injury after the contrast-enhanced computed tomography was conducted, following the 2018 Royal Australian and New Zealand College of Radiologists (RANZCR) guidelines, which recommended the development of written renal protection protocols, implementation of a checklist, and engagement with the local nephrology team [19]. In our clinical audit, we provided similar recommendations to those of a previous study [19]. We followed the guidelines published by Basildon and Thurrock University Hospitals NHS Foundation Trust instead of the RANZCR 2018 guidelines.

Limitations

It is important to note the limitations of our clinical audit. The study had a limited population as it was a clinical audit. The study was limited to an elderly hospital inpatient and outpatient population, excluding a healthier, younger (< 75 years) population and emergency patients. This limits the generalizability of our findings. Additionally, the study did not account for the type or amount of contrast media used, which are crucial factors influencing the risk of CIN. Lastly, the study was conducted only at the District Headquarters Hospital, not including our other affiliated institution, Mufti Mehmood Memorial Teaching Hospital, which could have provided a broader perspective on the adherence to renal protection protocols and hydration status.

## Conclusions

The clinical audit in District Headquarters Hospital, Dera Ismail Khan, successfully identified and addressed loopholes in hospital protocols related to renal function and hydration assessment before and after contrast CT. These improvements were achieved over three cycles. Continued education, regular monitoring, re-assessing, and expanding the audit to another hospital in Dera Ismail Khan will help maintain and increase patient care and safety standards, prevent CIN, and ensure better renal health of elderly patients. The medical teaching institute in Dera Ismail Khan has two hospitals. We initially conducted this audit in the District Headquarters Hospital, Dera Ismail Khan, and it will be expanded to Mufti Mehmood Memorial Teaching Hospital, Dera Ismail Khan.
